# Supplementary material for: Mechanism of MRX inhibition by Rif2 at telomeres
Source: Nat Commun. 2021 May 12;12:2763. doi: 10.1038/s41467-021-23035-w (PMC8115599; doi:10.1038/s41467-021-23035-w)
Supplement: Supplementary file 1 — Supplementary Information [file 41467_2021_23035_MOESM1_ESM.pdf]

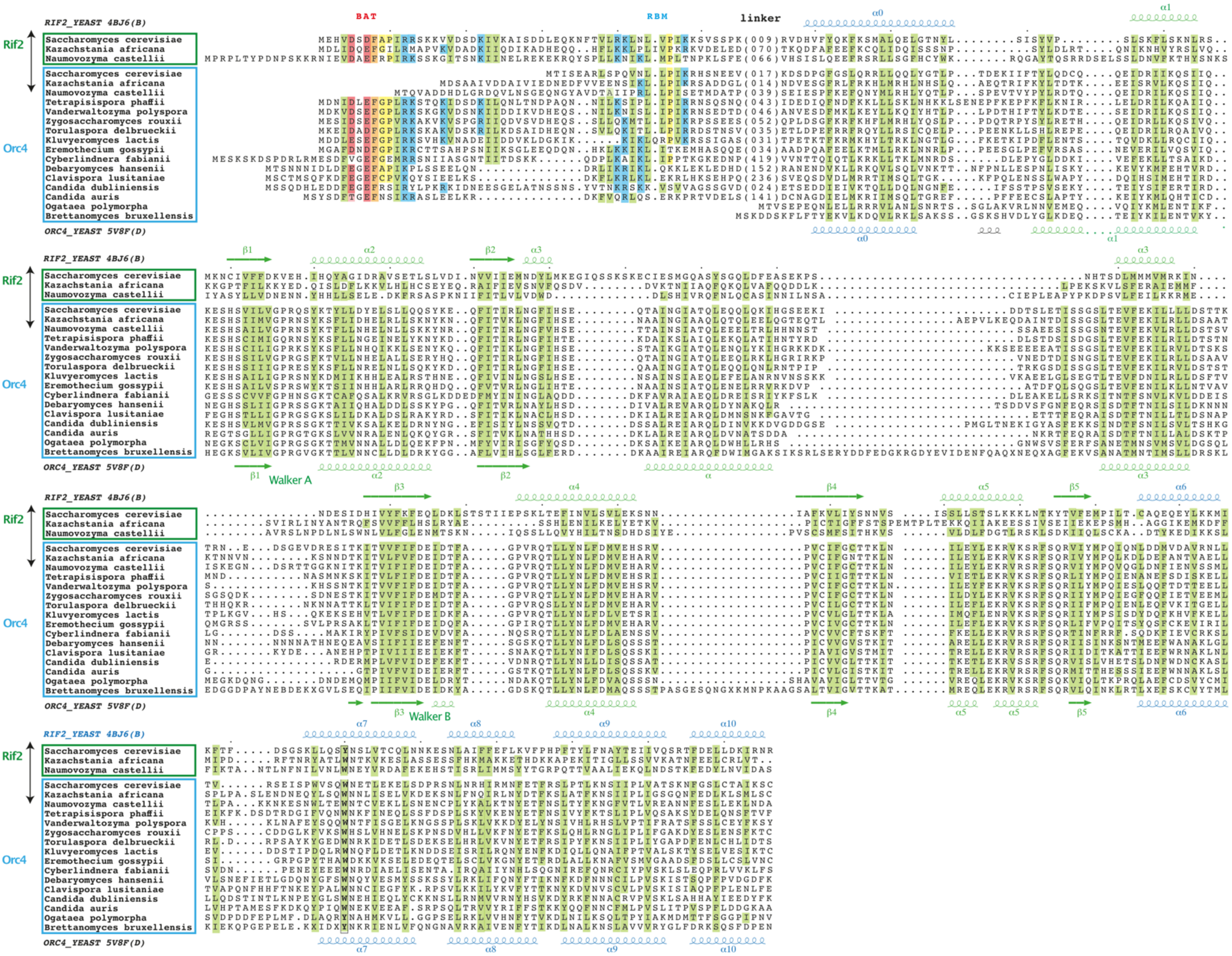

Supplementary Figure 1. Alignment of Rif2 and Orc4 from *Saccharomycetales* yeast species. Sequence Accession numbers : **Rif2 :** *Saccharomyces cerevisiae* : Q06208, *Kazachstania africana* : XP_003954885.1, *Naumovozyma_castellii* : XP_003677686.1, **Orc4:** *Saccharomyces cerevisiae* **:** P54791, *Kazachstania africana* : XP_003955485.1, *Naumovozyma_castellii* : XP_003674441.1, *Tetrapisispora_phaffii* : XP_003686307.1, *Vanderwaltozyma_polyspora* : XP_001643535.1, *Zygosaccharomyces_rouxii* :XP_002496500.1, *Torulaspora_delbrueckii* : XP_003683125.1, *Kluyveromyces_lactis* : XP_452959.1, *Eremothecium_gossypii* : NP_983126.1, *Cyberlindnera fabianii* : ONH65289.1, *Debaryomyces hansenii* : XP_459748.2, *Clavispora XP*_002615148.1, *Candida dubliniensis* XP_002420502.1, *Candida auris* : PIS52278.1, *Ogataea parapolymorpha* : XP_013933153.1, *Brettanomyces bruxellensis* : EIF47200.1

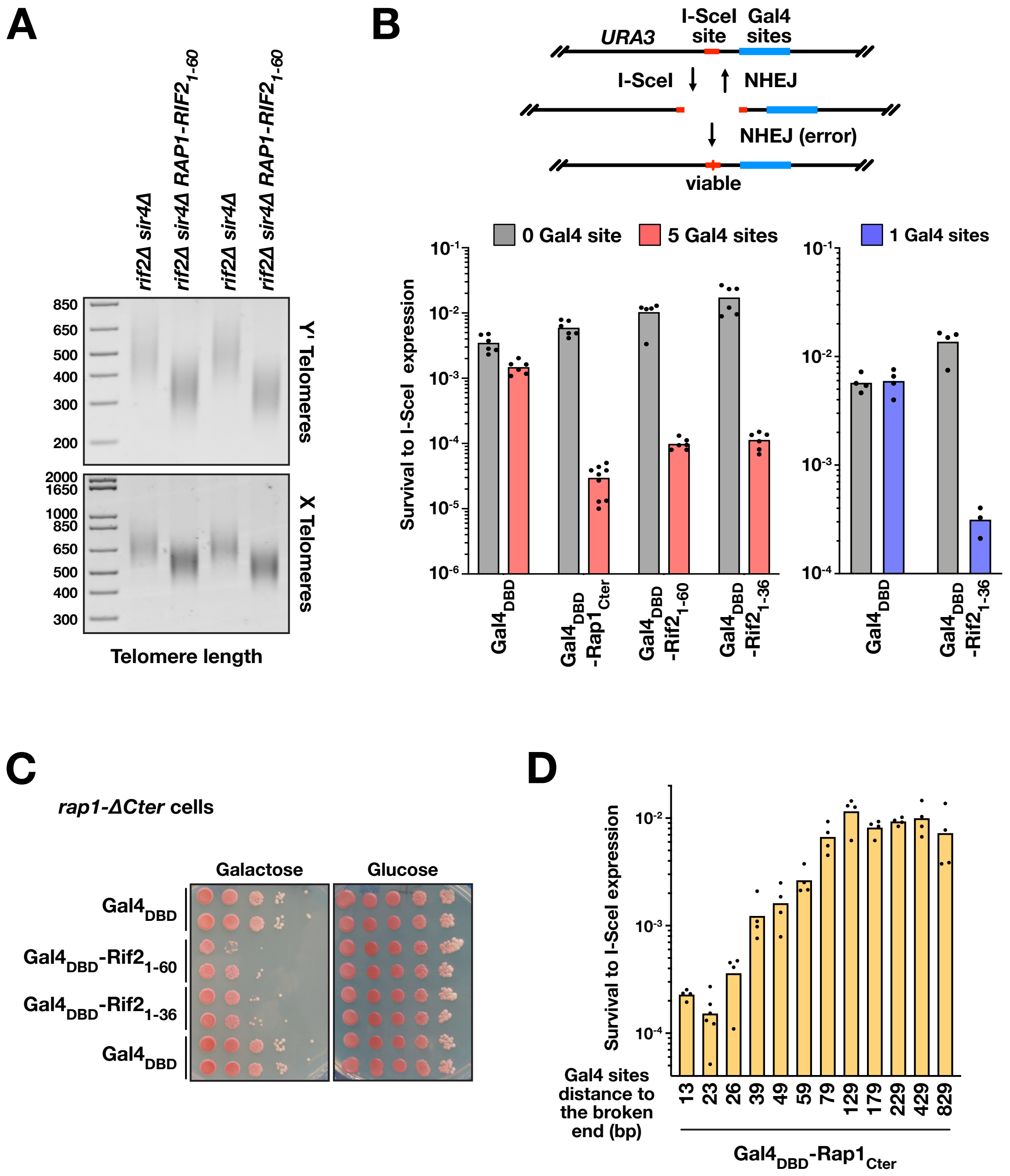

Supplementary Figure 2. (**A**) Fusing Rif2 N-terminal region to Rap1 C-terminal end (*RAP1-RIF2$_{1-60}$*) shortens telomere in cells lacking Rif2 and Sir4. Y' and X telomeres amplified by PCR. TG_{1-3} repeats length is the size of the amplified fragments minus 140 bp for Y' telomeres and minus 360 bp for X telomeres. (**B**) Top panel: single-site I-SceI assay used to estimate NHEJ efficiency. One I-SceI site is inserted downstream of the endogenous *URA3* gene. In survivors to continuous I-SceI expression, NHEJ-dependent mutations of the I-SceI site prevent further cleavage. Bottom panel: NHEJ inhibition by Rap1 C-terminal domain and Rif2 N-terminal region targeted at broken ends using the single-site I-SceI assay. Means from independent cell cultures. (**C**) NHEJ inhibition by Rif2 N-terminal region in cells lacking Rap1 C-terminal domain (*rap1-Δ670-807*) using the two-sites I-SceI assay. In this *rap1* mutant context, relative survival to I-SceI continuous expression is increased, perhaps a consequence of slow growth and of a reduced I-SceI expression. (**D**) Increasing distances between the broken end and the Gal4 binding sites decrease NHEJ inhibition by Rap1 C-terminal domain (two-sites I-SceI assay). Means from independent cell cultures.

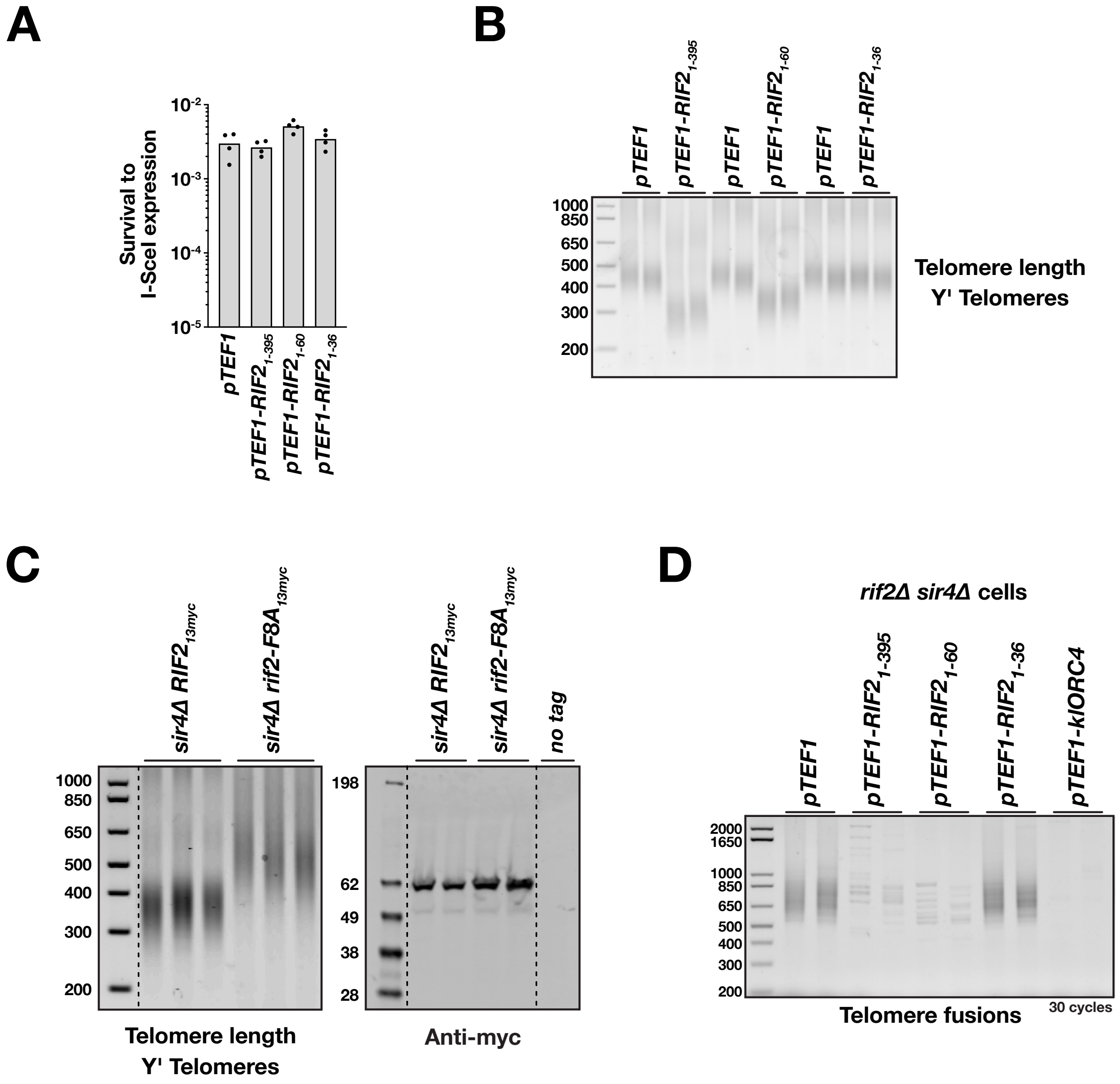

Supplementary Figure 3. (**A**) Rif2 overexpression does not challenge NHEJ at broken ends (two-sites I-SceI assay). Means from independent cell cultures. (**B**) Rif2 overexpression (full length and 1-60 fragment) shortens telomeres (Y' telomeres amplified by PCR). (**C**) Left panel: elongation in *rif2-F8A* cells lacking Sir4 (Y' telomeres amplified by PCR) (Kaizer *et al.* 2015). Right panel: the *rif2-F8A* mutation does not lower Rif2 protein level (9E10 anti-myc western blot) (Kaizer *et al.* 2015). (**D**) Rif2 (full length and 1-60 fragment) and klOrc4 overexpression protect telomeres against NHEJ-dependent fusions in cells lacking Rif2 and Sir4 (fusions between X and Y' telomeres).

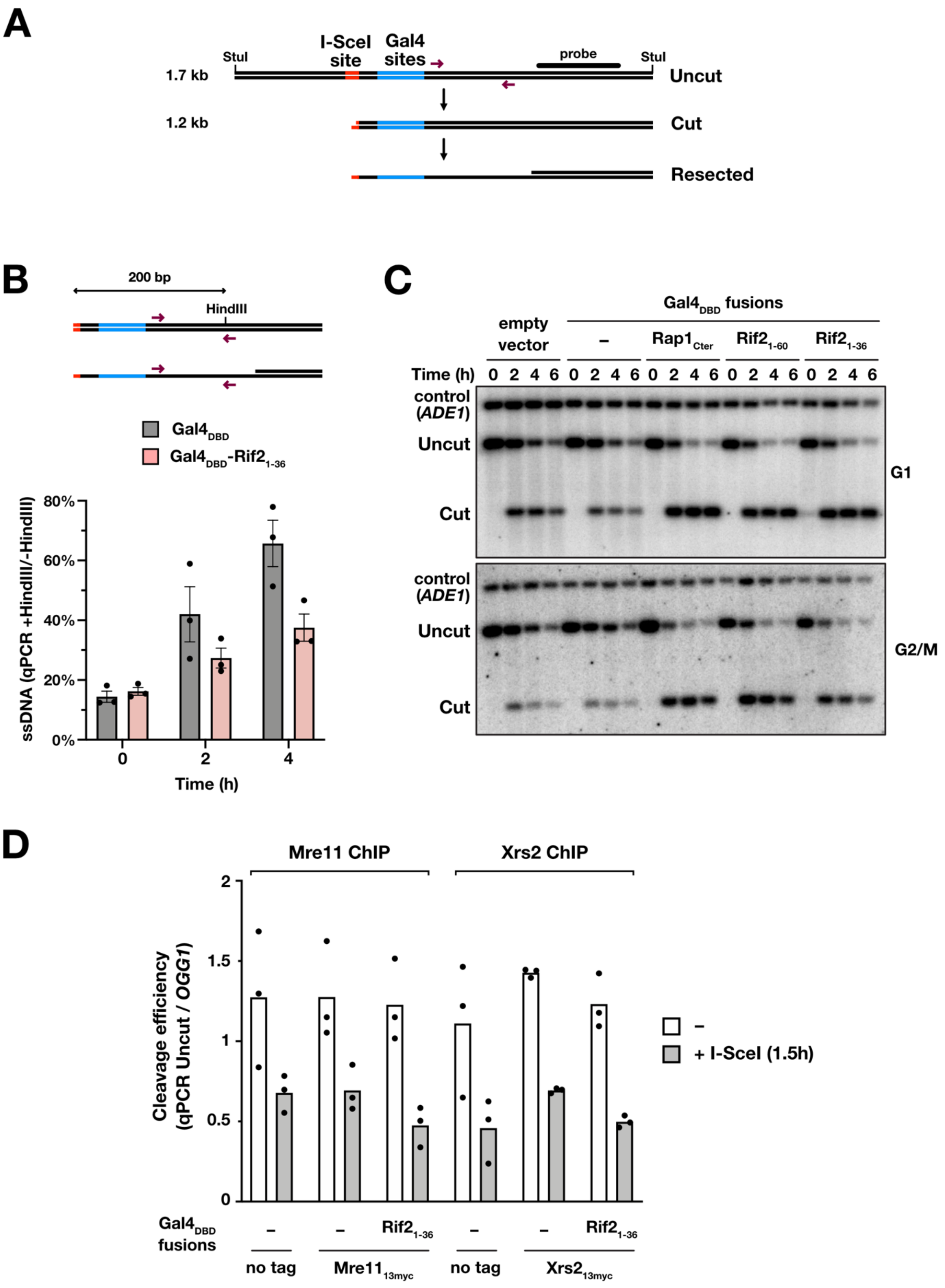

Supplementary Figure 4. (**A**) I-SceI resection assay and position of the primers used for the ChIP qPCR of the main Figure 3B (**B**) Single-strand (HindIII-resistant) quantification by qPCR (Zierhut and Diffley 2008 *EMBOJ* 27:1875). Means and SEM from independent samples. (**C**) Rap1 C-terminal domain and Rif2 N-terminal region stabilises broken ends in NHEJ-deficient cells lacking Lif1. The stability of I-SceI-induced broken ends with 5 Gal4 sites determined by Southern blot in G1 and/or G2/M arrested cells. (**D**) I-SceI cleavage efficiency in the ChIP samples of the main Figure 3B. Means from independent samples.

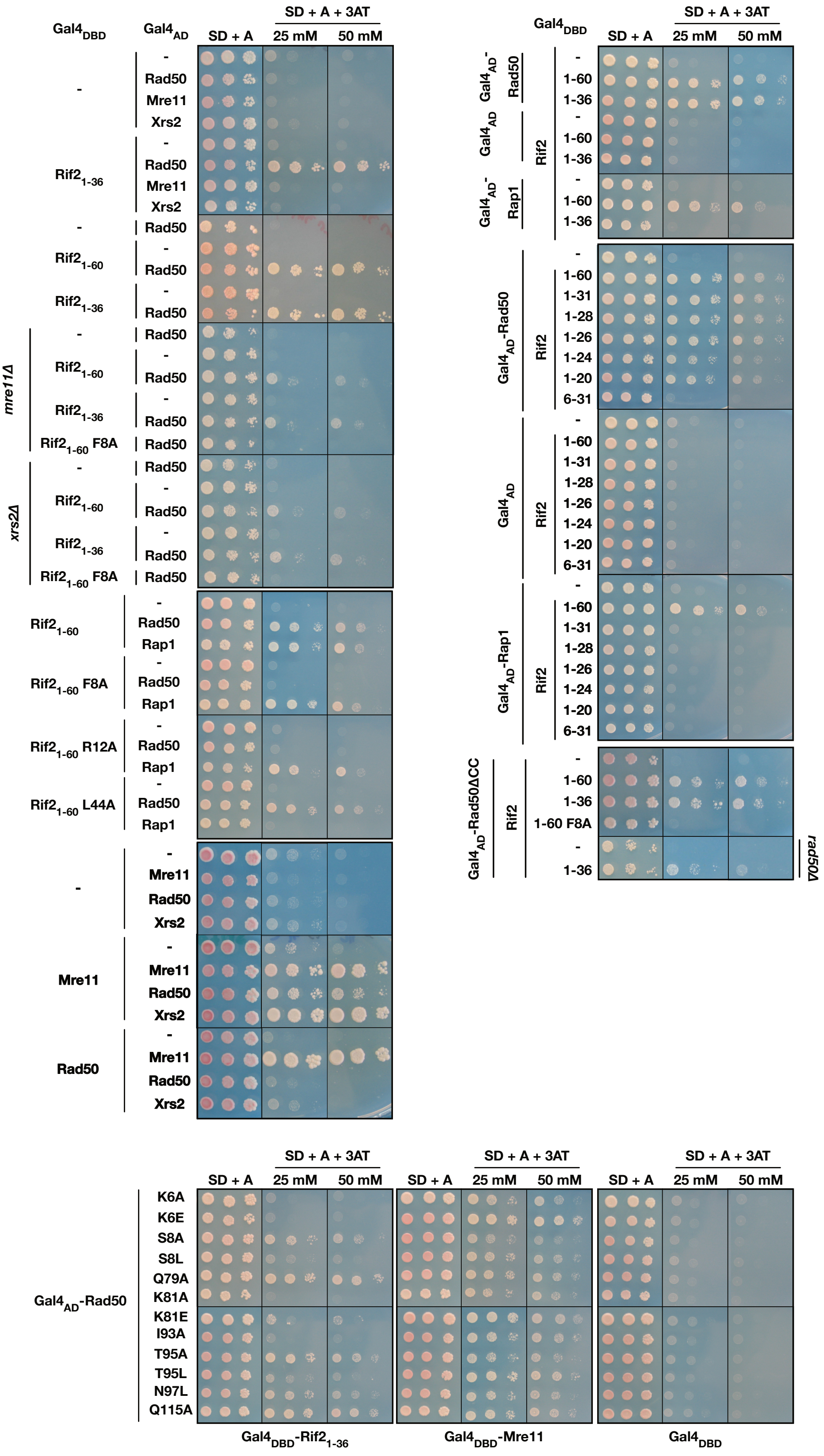

Supplementary Figure 5. 2-Hybrids interactions between Rif2 and Rad50. Drop assays on synthetic medium plates complemented with adenine and histidine (SD+A) and on synthetic medium plates complemented with adenine and His3-inhibtor 3AT (SD+A+3AT). 10-fold serial dilutions of saturated cultures.

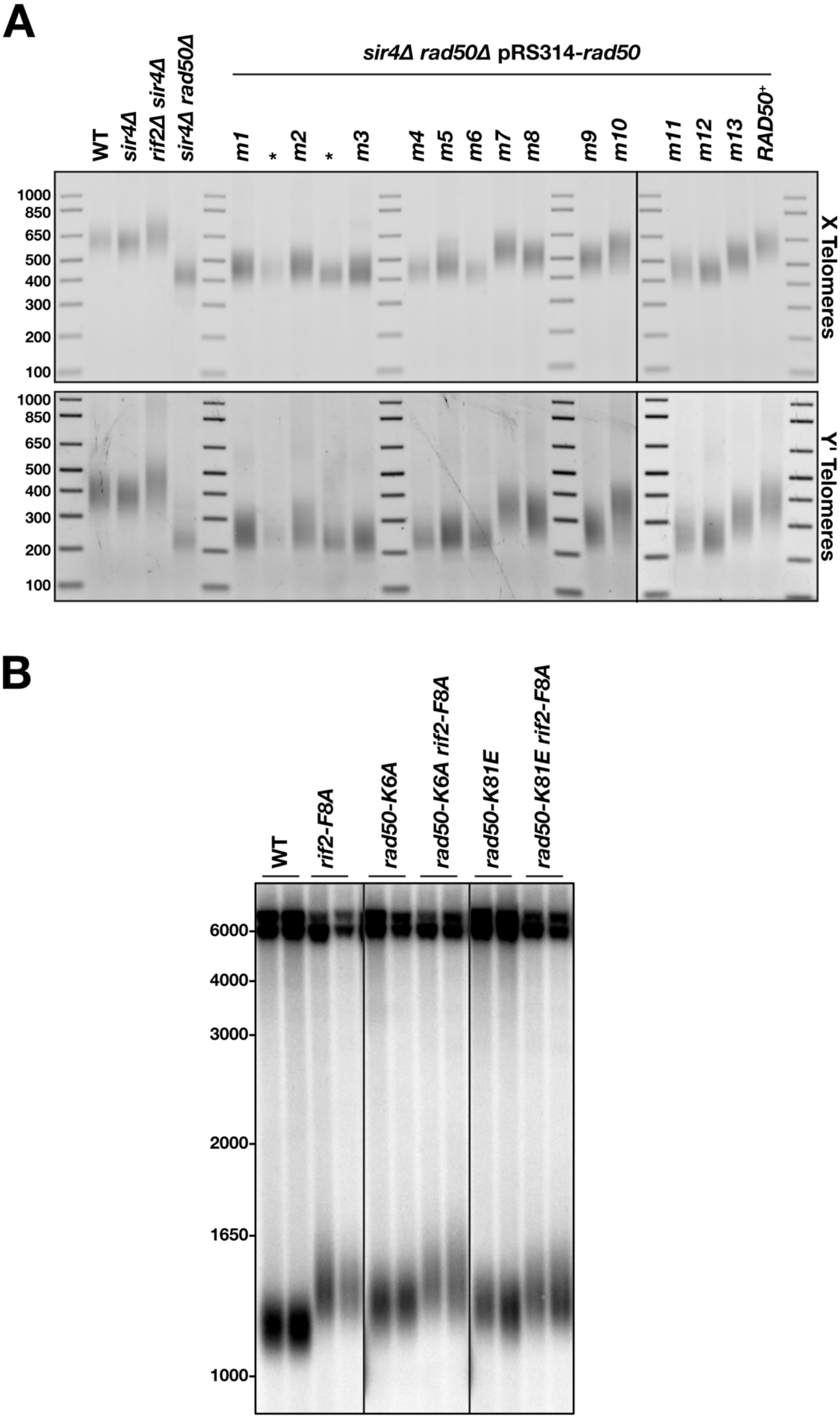

Supplementary Figure 6. (**A**) Telomere length phenotype of the screened *rad50* alleles increasing the frequency of chromosome fusions in cells lacking Sir4. Y' and X telomeres amplified by PCR. (**B**) Impact on telomere length of mutants *rad50-K6A* and *rad50-K81E* in cells harbouring the *rif2-F8A* allele (Southern blot, Y' probe, XhoI digest).

A

B

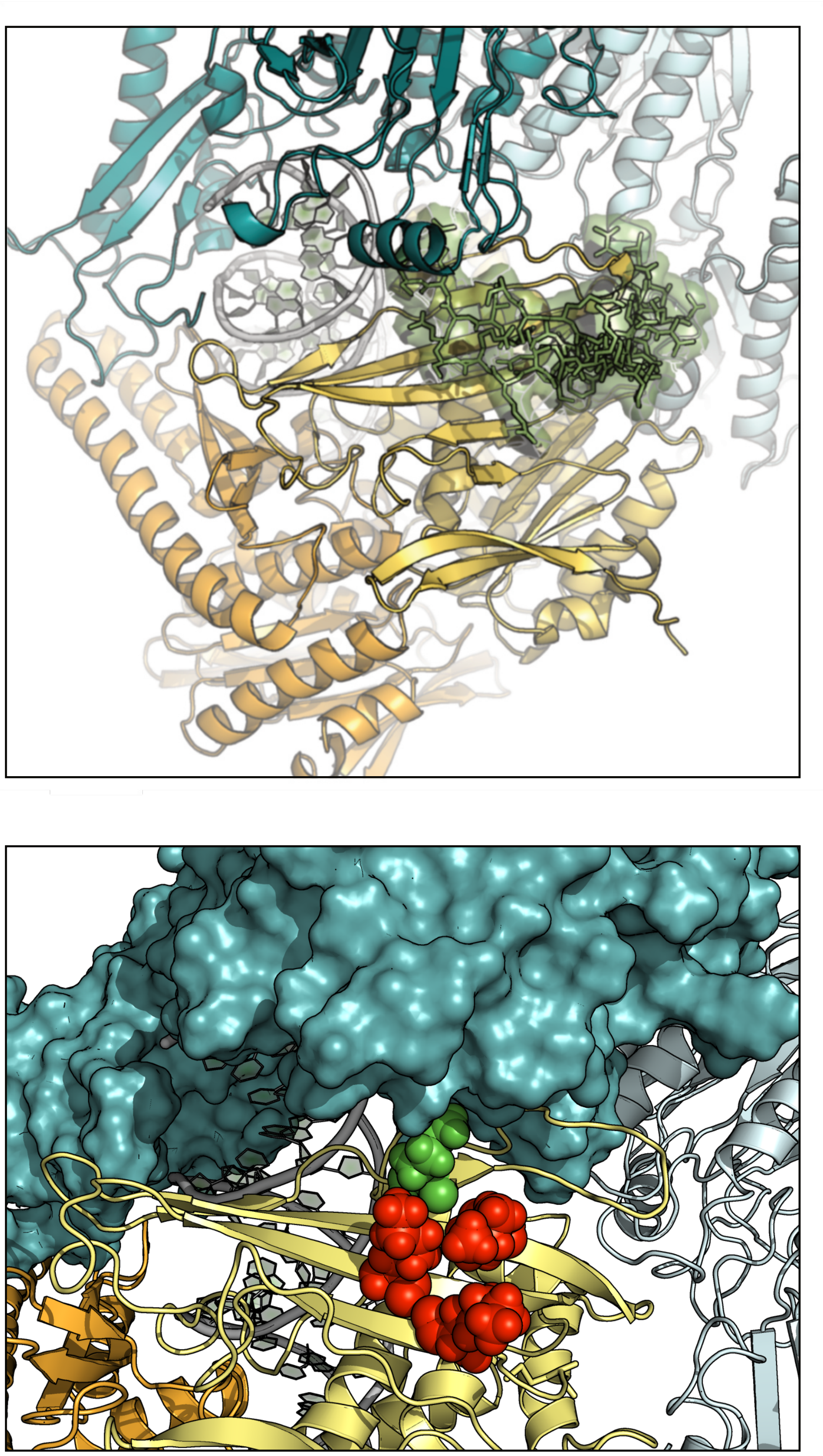

Supplementary Figure 7. (**A**) Envelope of the BAT core peptide (Rif2 residues 4-14, in green) belonging to the Haddock cluster 2 shown after superimposition of the Rad50 structures of the clusters on that of the DNA-bound Rad50-Mre11 complex model. (**B**) Models of *S. cerevisiae* Rad50-Mre11 complex in the DNA-bound active state (Rad50 orange/yellow, Mre11 teal/light blue). Rad50 Q115 residue highlighted in green. Rad50 residues K6, K81 and I93 highlighted in red.

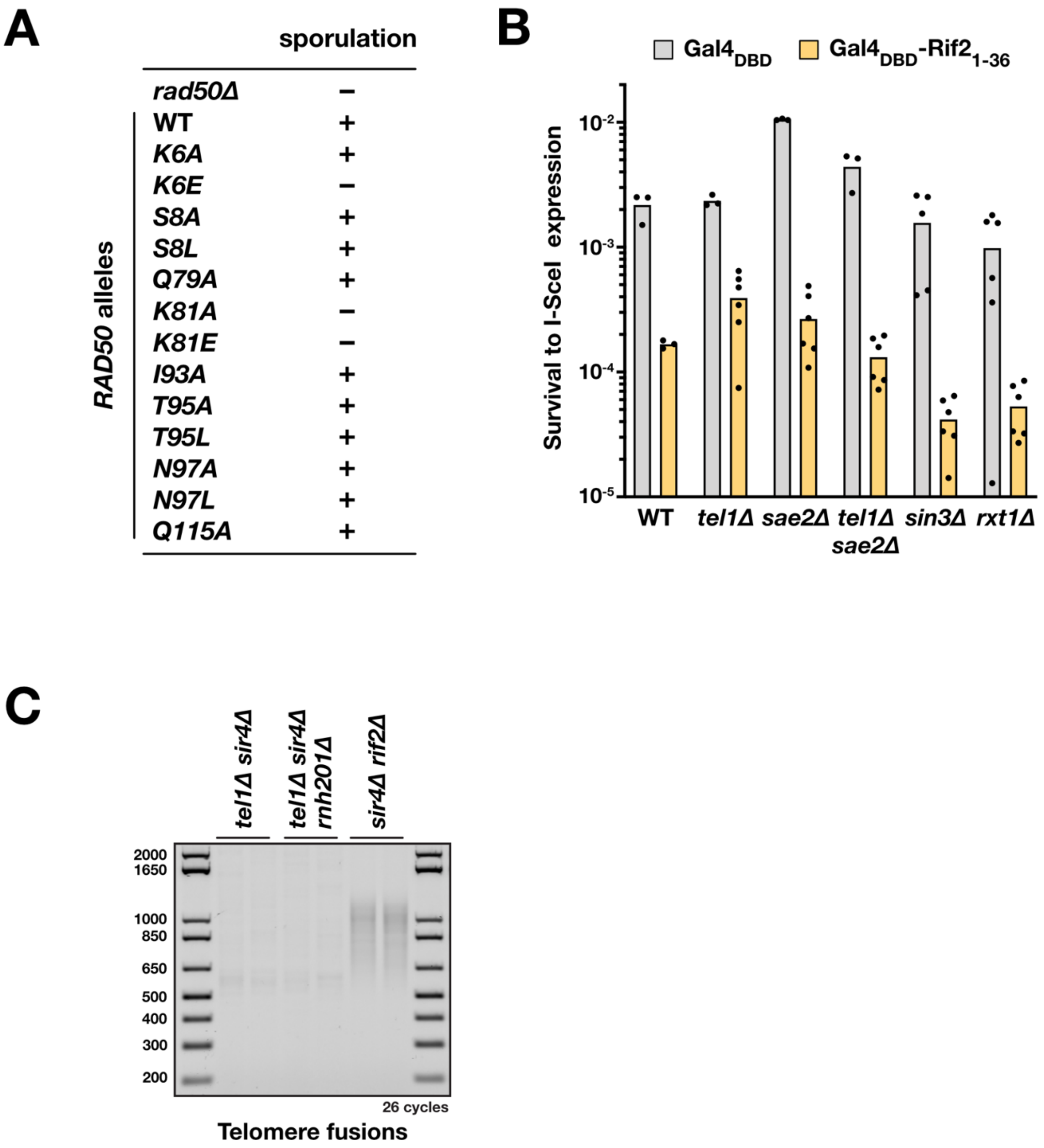

Supplementary Figure 8. (**A**) Sporulation of *rad50Δ/rad50Δ* diploid cells complemented with *rad50* alleles expressed from a *CEN/ARS* plasmid (pRS314). (**B**) Rif2 BAT motif inhibits NHEJ at broken ends in cells lacking Tel1, Sae2 and Rpd3L subunits Sin3 and Rxt1. Two-sites I-SceI assay. Means from independent cell cultures. (**C**) Rnh201 loss does not increase the frequency of telomere fusions in cells lacking Tel1 and Sir4. Fusions between X and Y' telomeres.
