# Supplementary material for: Mechanism of MRX inhibition by Rif2 at telomeres
Source: Nat Commun. 2021 May 12;12:2763. doi: 10.1038/s41467-021-23035-w (PMC8115599; doi:10.1038/s41467-021-23035-w)
Supplement: Supplementary file 3 — Description of Additional Supplementary Files [file 41467_2021_23035_MOESM3_ESM.pdf]

Description of Additional Supplementary Files

File Name: Supplementary Data 1
Description: List of strains, plasmids and primers used in this study.
